# BRCA mutations in the manifestation and treatment of ovarian cancer

**DOI:** 10.18632/oncotarget.18280

**Published:** 2017-05-30

**Authors:** Zimin Pan, Xing Xie

**Affiliations:** ^1^ Women's Hospital, School of Medicine, Zhejiang University, Hangzhou, Zhejiang Province, People's Republic of China

**Keywords:** BRCA, ovarian cancer, pathogenesis, chemotherapy, targeted therapy

## Abstract

*BRCA* genes are important for the integrity and stability of genetic material and play key roles in repairing DNA breaks via high fidelity homologous recombination. *BRCA* mutations are known to predispose carriers to gynecological malignancies, accounting for a majority of hereditary OC cases. Known to be lethal, OC is difficult to detect and control. Testing for *BRCA* mutations is a key step in the risk assessment, prognosis, treatment and prevention of OC and current clinical guidelines recommend *BRCA* mutation testing for all OCs of epithelial origin. Studies have established that ovarian tumors harboring *BRCA* mutations have distinct molecular and histo-pathological features that can be exploited for effective, targeted treatment. Deficiencies in DNA repair pathways that arise as a result of BRCA mutations make them hypersensitive to DNA-damaging treatments such as platinum chemotherapy and PARP inhibitors. Different combinations of treatment regimens which have the potential to greatly improve prognosis and disease outcomes are currently being evaluated. However, the issue of developing resistance to these treatments remains unresolved. This review emphasizes unique features of *BRCA* mutated OC and outlines the lay of the land in terms of diagnosis and treatment, while aiming to unravel the challenges that are part of its management.

## INTRODUCTION

Ovarian cancer (OC) is known to cause the largest number of deaths among cancers of gynecologic origin. Inheritable mutations increase the risk of development of OC in carriers [1, 2]. Dominant, autosomally transmitted hereditary OC arises from mutations in 2 key types of genes: the *BRCA* (breast cancer susceptibility) genes (called hereditary breast and OC [HBOC] syndrome) and the *MMR* (DNA *mismatch repair)* genes (called hereditary non-polyposis colorectal cancer [HNPCC] syndrome or Lynch II syndrome) [1]. Both sets of genes are involved in the repair of genetic lesions. However, of these two syndromes, mutations in the *BRCA* 1/2 (*BRCA*) genes responsible for HBOC syndrome are more common, accounting for 90% of all the hereditary OC cases [3]. The *BRCA* genes are tumor suppressors, and a mutation in either one is known to predispose carriers to several types of cancer [4, 5].

Although they have been associated with an increased risk of occurrence of various other types of cancer that are not of gynecologic origin, the majority of specific inherited mutations in *BRCA1* and *BRCA2* increase the risk of female breast and ovarian cancers. Together, inheritable mutations in *BRCA1* and *BRCA2* account for around 15% of OC cases. While approximately 1.3% of women in the general population are likely to develop OC, recent estimates suggest that about 40%-60% of women who inherit a *BRCA1* mutation and between 11% and 27% of women who inherit a *BRCA2* mutation will develop OC by 80 years of age, with the risk of development of OC being higher in *BRCA1* carriers [6–10]. Thus, a mutation in either gene represents a significantly increased risk of OC in women [6]. The histopathology and molecular characteristics of OC that occur as a result of *BRCA* mutations are distinct from other types of ovarian malignancies, and several studies have also demonstrated that these features make such tumors more responsive to certain types of treatment regimens [11, 12]. Despite this, such tumors are hard to detect. The purpose of this review is to emphasize the recent advances in *BRCA* 1/2 linked OC prevention, detection, and treatment. In addition, it also highlights the possible areas of future investigation that have the potential to improve patient health and disease outcomes.

### The BRCA genes

*BRCA1* and *BRCA2* are located on chromosomes 13 (13q12.3) and 17 (17q21), respectively. Both genes occupy very large regions of the human genome and span about 70 kb of DNA. *BRCA1* contains 22 exons and *BRCA2* contains 27 exons and both encode multi-domain proteins. Their primary sequences are rich in repetitive DNA elements [13–15]. They are broadly classified under the umbrella of tumor suppressors and are involved in cellular pathways for the repair of genetic lesions. In addition, a loss of function of either gene results in similar physiological consequences and also increases the risk of developing similar types of cancer, primarily breast and ovarian. However, both molecules are distinct in terms of genetic sequence and perform a non-redundant molecular function [13].

Many studies have focused on studying these genes in detail, in order to identify mutations that result in a loss of protein function and thus in an elevated risk of malignancy. Studies have also focused on classifying mutations based on the genetic location in which they occur, and mutations in specific regions of both genes are known to predispose carriers to certain types of malignancies. The ovarian cancer cluster region (OCCR) of *BRCA2* has been well defined and studies have shown that mutations outside of the OCCR predispose carriers to malignancies that are not of ovarian origin. Conversely, mutations that lie within the OCCR of *BRCA2* predispose carriers to a significantly elevated risk of ovarian cancer compared with other malignancies. Studies have also shown that mutations in the 3’ region of the *BRCA1* gene are linked with a lower risk of OC, whereas mutations in regions further downstream are linked with an elevated risk of OC [16, 17]. Although the results of such studies are useful in estimating the risk of occurrence of certain kinds of malignancies in patients, they are not without exception and therefore must be interpreted with caution [18].

### Epidemiology and prevalence of BRCA mutations in OC

Over the past 2 decades since their discovery and cloning, *BRCA* mutation studies have been conducted in various populations across the world. These studies show that the highest prevalence of *BRCA* mutations in OC is in women with the serous form of epithelial OC. Generally, mutations exist in either *BRCA1* or *BRCA2* and rarely does the same person have mutations in both the genes. Mutations in *BRCA* may be inherited within certain families and of germline origin or they may occur as tumor-only, non-inheritable somatic mutations in certain individuals [19]. Either germline or somatic mutations in *BRCA* account for 20% of all the OCs. The germline mutation rate in these genes is currently estimated at about 15% in OC cases, whereas somatic mutations in the *BRCA* genes account for about 5% [20–22].

The prevalence of inherited germline *BRCA* mutations in different populations is highly variable and dependent on ethnicity. The highest prevalence of germline *BRCA* mutations exists in women of Ashkenazi Jewish origin. Norwegian, Danish, Icelandic people, and people of French Canadian descent were also found to have a higher incidence of *BRCA* mutations [23–25]. In such closed populations, certain identifiable, specific mutations in the *BRCA* genes exist and these are known as founder mutations. For example, the 185delAG and 5382insC mutation in the *BRCA1* gene and the 6174delT mutation in the *BRCA2* gene are the known founder mutations that exist in Ashkenazi Jewish women [9, 26]. The prevalence of *BRCA* mutations in the Chinese population is similar to that in Western countries; however, a recent study found that the spectrum of mutations observed in Chinese populations differs from Western counterparts [27]. Another study also identified novel founder mutations present in populations from Eastern China [28]. In a multicenter BRCAm prevalence study conducted in 2016, Prof. Wu (Fudan University Shanghai Cancer Center) reported that the observed *BRCAm* prevalence was 28.5%, which is higher than what was found in other studies. This difference may be attributed to a higher percentage of Chinese ovarian cancer patients with high grade serous and late stage ovarian tumors observed in this group [29].

### Pathogenesis, pathophysiology, and molecular characteristics of OC linked to BRCA mutations

Epithelial ovarian cancer (EOC) is classified as serous, mucinous, endometrioid, clear cell, or undifferentiated carcinoma etc., depending on the histological cell type. Of these, the first 4 are predominant in EOC [30]. The pathogenesis of EOC determines the type under which it is classified, with type I EOC developing through the low-grade pathway and type II EOC developing through the high-grade pathway. The main difference between the 2 types is that type I cancers show a spectrum of biogenesis from benign to malignant, whereas high-grade type II cancers arise de novo as aggressive neoplasms. Histological investigations of ovarian tissues obtained after preventive oophorectomy on *BRCA* mutation carriers were compared with those without *BRCA* mutations, and the only discernible difference was a higher frequency of surface micropapillae, the clinical significance of which has not been established [31]. As a consequence of these factors, there is no identifiable pre-cancerous lesion in type II carcinomas, and they are therefore detected at a later stage of progression [32, 33]. Although the claim needs to be supported by further investigation, there is preliminary evidence to show that fimbral dysplasia may be the pre-cancerous lesion for serous high-grade carcinomas [34, 35]. Fortunately, high-grade tumors have also been found to be largely chemo-sensitive. It is of particular interest that currently, type II disease is the predominantly occurring one in diverse populations.

Most OCs linked to *BRCA* mutations are high-grade, serous epithelial OCs (HGSOCs) and *BRCA* mutations are less likely to predispose carriers to other classes of EOC such as mucinous, endometrioid, or clear cell EOC [36–38]. BRCA mutations are present in more than one fifth of all high-grade serous OC cases [39]. However, *BRCA* mutations are purported to account for between 5%–15% of endometrioid and clear cell subtypes of OC [18, 40].

In addition, patients with BRCAm OC show a much higher incidence of HGSOC than patients having sporadic OC, and the majority of BRCAm OC-linked HGSOC tumors have been shown to arise in the fallopian tube [41–43]. Thus, as mentioned above, although the majority of BRCA-linked OC cases are of the high grade serous type, BRCAm OC also manifests as other histological sub-types, making *BRCA* testing an important tool for the effective treatment and resolution of various types of EOC [44]. *BRCA* mutation testing is also recommended by the NCCN guidelines as an important prerequisite for all patients presenting with EOC [45, 46]. At the tissue level, *BRCA*-linked cancers also generally show an increased tumor infiltration of immune cells. These factors are probably linked to the favorable prognostic outcome in *BRCA* mutated cancers compared with those cancers that are not a result of *BRCA* dysfunction [47]. Several studies have also shown that there is an increased frequency of p53 mutations and p53 overexpression in OCs arising from *BRCA* mutations compared with those that arise sporadically [37, 47, 48]. Analysis of EOC tissues has also shown that the homeobox gene *HOX9* is up-regulated and this results in a more permissive environment for tumor growth and development through its effect on the differentiation of Cancer associated fibroblasts (CAF) [49]. In addition, other HOX genes have also been shown to be differentially expressed, with maximum genetic dysregulation occurring in case of HGSOC, the histological subtype that BRCA mutations are largely responsible for. Also, it has been shown that different HOX genes are dysregulated depending on whether the tumor is platinum sensitive or resistant and it is possible that this ties back to the BRCA mutation status of the tumor [50]. In contrast to breast cancers arising from *BRCA* mutations, HER2 expression in BRCAm OC have not been found to be significantly up-regulated compared with controls, although there are some studies that report an increase in HER2 expression in certain cases of HBOC.

### Treatment and prognosis of OC caused by BRCA mutations

Although *BRCA* carriers are more likely to develop OC, they respond better to certain chemotherapy regimens and to some types of targeted treatment, notably platinum-based chemotherapy and poly-ADP ribose polymerase (PARP) inhibition. This augmentation in response rate can be attributed to the DNA-damaging effects of the treatment regimens that exploit the molecular and phenotypic characteristics of *BRCA*-linked tumors. Recently, it has been established that these molecular and phenotypic characteristics are not restricted to *BRCA*-linked tumors, but have also been found in other cancers that have a dysfunction in DNA repair genes. Thus, such characteristics are said to confer a *BRCAness* phenotype on the tumor and the benefits of DNA-damaging therapy can be extended and used to treat such tumors as well. This section of the review aims to characterize the responses of *BRCA*-linked tumors to different treatment regimens, since they can be extrapolated and adapted for the treatment of a larger population.

Several studies have reported that short-term prognosis and progression-free survival (PFS) in patients with BRCAm OC are better than in patients with OC because of non-*BRCA*–linked sporadic mutations in response to various treatment regimens [51, 52]. Moreover, because of improved prognosis in patients with *BRCA* dysfunction, VEGFR3 inhibition is being developed as a treatment to induce low levels of *BRCA* 1/2 in patients with sporadic OC or in patients who originally presented with BRCAm OC but experienced a *BRCA* gene reversion [53]. Despite these positive outcomes for patients with BRCAm OC, the issue of overall survival (OS) rates in patients with BRCAm OC versus control participants is controversial. A very early study that compared OS rates between control participants and patients with BRCAm OC by Ruben et al reported a significantly higher OS for patients with BRCAm OC [52]. However, since then, many studies that undertook similar investigations have reported no significant difference between OS rates in patients with BRCAm OC versus control participants [51].

A study in a cohort of Jewish women suggested that OC patients with germline *BRCA* mutations showed a better prognosis (survival period of 91 months and disease free interval of 49 months) than OC patients with somatic mutations (survival period of 54 months and disease free interval of 19 months) [54]. In addition, the results of a retrospective study suggested that OC patients with a mutation in *BRCA2* (HR = 0.20) are likely to have higher PFS rates than OC patients with a mutation in *BRCA1* (HR = 0.70) or no *BRCA*-related dysfunction [3, 55]. However, this conclusion was from an isolated study and conflicts with the results of a recent meta-analyses of 14 studies which suggested that both, *BRCA1 and BRCA2* mutation status were equal predictors of a better OS rate (pooled HR of 0.65 in *BRCA1* mutation carriers and a pooled HR of 0.61 in BRCA2 mutation carriers) in Ovarian Cancer patients [56]. Interestingly, this result was less convincing when mutations in the *BRCA1* promoter region were analyzed, indicating that there might be differences in the course of treatment required for patient subgroups having mutations in different regions of the gene [57]. Presented below (and summarized in Table 1) is a discussion on the currently available options for the treatment of BRCAm OC.

**Table 1:** Summary of different treatments and outcomes in patients with OC based on *BRCA* mutation status

References	No. of patients	Type of OC	Treatment	*BRCA* mutation status	PFS	OS
Adams SF et al, 2011 [58]	23	EOC	(PLD) Doxil	*BRCA* 1/2 positive versus sporadic OC	27.1 weeks versus 17 weeks	89.1 weeks versus 48.3 weeks
Ledermann JA et al, 2012 [59]; Ledermann JA et al, 2014 [60] (Aka Study 19- basis for BRAC Analysis approval)	254	PSR HGSOC	Olaparib maintenance therapy versus placebo	*BRCA* 1/2 positive	11.2 months versus 4.1 months	No difference reported
Oza AM et al, 2015 [61]	107	PSR HGSOC	Paclitaxel + carboplatin versus paclitaxel + carboplatin + olaparib maintenance	*BRCA* 1/2 positive versus *BRCA* 1/2 negative	9.6 versus 12.2 months	Not reported
Louroso D et al, 2016 [11]	100	PSR OC versus PRR OC	Trabectedin	*BRCA* 1/2 positive or BRCA*ness* versus unreported	No difference w.r.t. *BRCA* status	No difference w.r.t. *BRCA* status
Monk BJ et al, 2015 [62]	41	PRR OC	Trabectedin + PLD versus PLD	*BRCA* 1/2 positive	13.5 versus 5.5 months	23.8 versus 12.5 months
Liu JF et al, 2014 [63]	90	PSR HGSOC	Olaparib + cediranib versus olaparib	Mixed: *BRCA* positive + unknown	17.7 versus 9 months	Not reported

OC: Ovarian Cancer; BRCA: Breast cancer, early onset; PFS: Progression free survival; OS: Overall Survival; EOC: Epithelial OC; PSR HGSOC: Platinum Sensitive Recurrent High Grade Serous OC; PRR OC: Platinum resistant/refractory OC; PLD: Pegylated liposomal doxorubicin.

### Surgical intervention

Surgical cyto-reduction and de-bulking of the tumor is largely considered the first line of treatment in patients who develop OC. Generally, bilateral salpingo-oophorectomy is recommended, and depending on the stage of the tumor and the age of the patient, a hysterectomy may also be recommended. In general, studies have reported an equal de-bulking rate in patients with BRCAm OC versus patients with sporadic OC, with no specific advantage for *BRCA* mutation carriers in terms of reduction in tumor size after surgery.

### Chemotherapy

As in the case of other cancers, chemotherapy is a commonly used treatment in the resolution of BRCAm OC. However, systemic chemotherapy presents issues of high toxicity. It is well established that BRCAm OC tumors respond better to DNA-damaging agents, and therefore platinum-based DNA-damaging chemotherapy is a commonly recommended course of treatment for BRCAm OC.

### Platinum-based chemotherapy

Platinum-based chemotherapy, either cisplatin or carboplatin, is commonly used in the treatment of OC. Those malignancies that have been treated with platinum-based therapies as a first line of treatment but worsen or recur after 12 months of initial treatment or do not worsen or recur at all are termed platinum sensitive. Those that progress within 6 months of initial treatment are termed platinum resistant and those progressing between 6-12 months of platinum therapy are called partially platinum sensitive. A platinum-paclitaxel combination is generally used as the first line of treatment for OC [64]. Ovarian tumors of *BRCA* origin are more sensitive to platinum-based chemotherapy than sporadic OC cases. Several studies have shown that patients with *BRCA* mutations of either germline or somatic origin respond better to platinum-based chemotherapeutic regimens and demonstrate better prognosis and improved survival rates over a median range [65, 66]. However, studies have also shown that in certain cases, previously platinum-sensitive BRCAm OC can become resistant to platinum treatment. Analysis has revealed that this switch is likely to occur due to a reversion of the *BRCA* mutation that restores protein function in tumor cells [67, 68].

### Intra-peritoneal chemotherapy

The selected chemotherapy regimen may either be administered through an intravenous (IV) or intra-peritoneal (IP) route or by using a combination of the two depending on the treatment type and stage of presentation with OC. Currently, the standard of care for patients with EOC sometimes involves the administration of platinum-based chemotherapy and paclitaxel through a combination of routes, that is, either IV or IP. This combined model of treatment has been shown to be associated with a lower risk of death in patients for whom de-bulking surgery was effective [69]. However, such IP treatment causes very high toxicity within the peritoneal cavity.

A limited number of studies have assessed the use of IP chemotherapy in *BRCA* mutation positive versus *BRCA* mutation negative EOC. While studies have shown an improved PFS rate in *BRCA* mutation positive OC patients upon the use of platinum based IP chemotherapy (mainly cisplatin and paclitaxel) after surgical cyto-reduction, conclusive, direct evaluations of treatment efficacy between the IP and IV route based on *BRCA* mutation status are yet to be conducted [12, 70, 71]. Only one Phase III study, i.e., COG-172 has evaluated patient prognosis parameters after IP or IV chemotherapy based on BRCA mutation status. The results showed a better PFS in BRCA mutation positive patients treated with platinum based chemotherapy administered via the IP route, with a clinically insignificant effect on OS [72].

### Pegylated liposomal doxorubicin

Pegylated liposomal doxorubicin (PLD) is a treatment that was initially approved for the recurrent form of epithelial OC in patients in whom platinum-based chemotherapy failed [73]. The formulation increases the concentration of doxorubicin specifically in tumor tissues, and thus shows a lower toxicity profile than platinum-based chemotherapy. Compared with only doxorubicin, a significantly lower number of severe adverse events (SAEs) occur, especially in terms of adverse cardiovascular events. In a recent study that investigated the use of PARPi versus PLD in BRCAm OC, there was an unexpectedly high PFS demonstrated in the PLD arm of the trial, implying that PLD may be prescribed in future as a treatment that is specifically efficacious in *BRCA* mutation carriers [58, 74].

### Neo-adjuvant chemotherapy

In most OC cases, de-bulking surgery is recommended before the administration of chemotherapeutic or targeted treatment regimens to facilitate a reduction in the size of the primary tumor. However, in certain cases where the primary tumor is bulky, neo-adjuvant chemotherapy is recommended before surgical resection of the tumor. Neo-adjuvant chemotherapy can be used before de-bulking surgery to reduce the size of the primary tumor in patients presenting with stage III-IV EOC. In the CHORUS trial, the first line of treatment used was neo-adjuvant therapy, followed by surgery and standard chemotherapy. This was compared with the standard treatment regimen using first de-bulking surgery followed by chemotherapy. The investigators found that neo-adjuvant therapy was non-inferior and that there was no residual disease in the neo-adjuvant arm of treatment. However, this did not translate to an overall higher OS rate, and current research is focused on elucidating the biological mechanisms underlying this paradox [75].

Additionally, the efficacy of neo-adjuvant chemotherapy versus standard surgical de-bulking (prior to the administration of the chemotherapeutic regimen) based on *BRCA* mutation status remains to be evaluated. The study by Gorodnova *et al.*, (conducted in Slovic patients) showed that the majority of *BRCA* mutation carriers had a complete clinical response to platinum-based neo-adjuvant chemotherapy, while only a small percentage of patients who were negative for BRCA mutations responded favorably [76]. However, this study did not provide a head to head evaluation between the standard mode of chemotherapy administration versus the use of neo-adjuvant therapy, and the enhanced response of BRCAm OC patients could also be attributed to the use of a platinum based regimen rather than to the mode of chemotherapy administration.

### Targeted therapy

Aim: Improved efficacy and decreased toxicity treatment that exploits specific cellular and molecular characteristics linked to the origin of the cancer.

### PARP inhibitors

**Figure 1:**
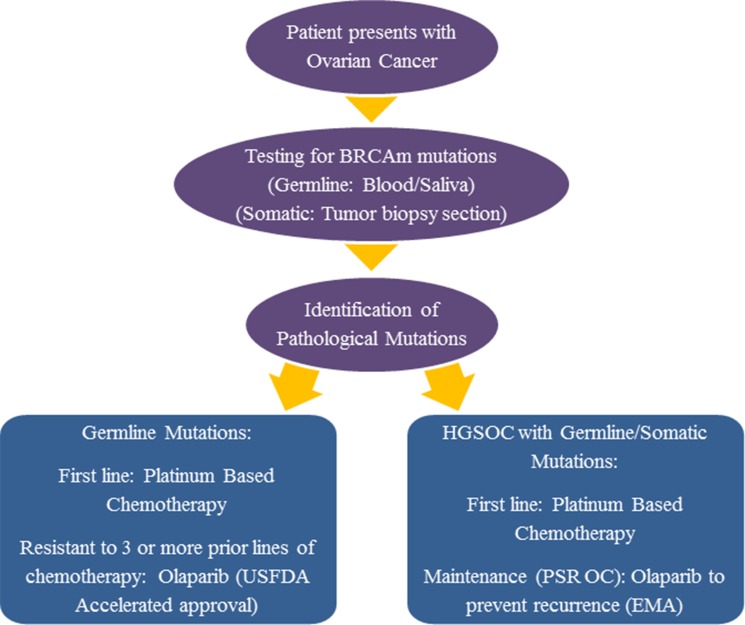
Incorporation of olaparib into the treatment regimen for BRCAm OC: USFDA versus EMA approval Outline of the testing and treatment procedure for patients presenting to the clinic with Ovarian Cancer. A comparison of the approval status for PARPi (Olaparib) in Europe versus America has been described. While the FDA has approved the use of Olaparib in patients with OC that failed to respond to three or more lines of chemotherapy, the EMA has approved Olaparib as maintenance treatment for PSR OC.

PARP inhibitors target the cellular enzymes PARP-1 and PARP-2 and thereby obstruct important molecular events necessary for effective DNA repair in the cell. Currently, there are numerous cellular pathways that they have been shown to interrupt. There are several hypotheses that attempt to explain the enhanced responsiveness of *BRCA*-deficient tumors to PARP inhibition. The most convincing of these is that they force the already *BRCA*-deficient cells to rely on HR as a repair mechanism, rendering them incapable of dealing with genetic lesions. This is known as synthetic lethality [77]. The current hypothesis for their improved effectiveness in treating OC resulting from *BRCA* mutations is linked to this fact. It was demonstrated in an open-label, non-randomized, phase II study that BRCAm OC is associated with a better response to apoptosis-inducing PARP inhibitors [78]. The proof of concept for the treatment of BRCAm OC with PARPi arose when laboratory tests showed that *BRCA*-deficient cells were extremely sensitive to PARP inhibition [77, 79]. In 2014, the FDA (Food and Drug Administration) and EMA (European Medicines Agency) approved the use of PARP inhibitor olaparib (AZD2281) for BRCAm OC. However, the setting for approval in both cases was different: the FDA approval was in the context of relapsing BRCAm OC and EMA approval was in the setting of maintenance treatment for BRCAm OC [80]. While the FDA approved the use of Olaparib for those gBRCA mutated Ovarian tumors resistant to at least three lines of prior chemotherapy, the EMA approved its use as maintenance therapy for HGSOC treated with first line chemotherapy. The 2017 NCCN guidelines for Ovarian Cancer are in line with the FDA approval for Olaparib. However, the NCCN panel does not recommend the use of Olaparib as maintenance therapy as the general consensus among experts was that a lack of evidence exists to support its use in this context (https://www.nccn.org). Olaparib was shown to be an effective course of monotherapy against advanced stage, relapsing BRCAm OCs with an ORR of 41%, leading to breakthrough approval that was granted by the FDA in 2014 [81]. Figure 1 provides a summary of the use of olaparib in the treatment of BRCAm OC in the context of FDA and EMA approval.

### Anti-angiogenic treatment

Aggressive angiogenesis is one of the defining features of malignant tumors. Cancerous cells show an increased rate of blood vessel production compared with normal cells, and VEGF and VEGFR are molecules that are highly expressed during the process of angiogenesis. VEGF is secreted by cancer cells and stimulates endothelial cells by binding to the tyrosine kinase receptor VEGFR on their surface, activating an intracellular cascade that ultimately results in an increased production of blood vessels [82]. Many treatments have focused on targeting this molecular interaction and this can be achieved by using either antibodies that target VEGF (the ligand) or those that target VEGFR (the receptor). Cediranib (AZD2171) is one such molecule that is currently being studied to retard the rate of angiogenesis in BRCAm OC by inhibiting the activity of VEGFR. A phase III study evaluated its use in recurrent platinum-sensitive EOC in combination with chemotherapy and as maintenance therapy and found that although progression-free survival in patients improved, increased toxicity was still a concern [83]. Bevacizumab is a humanized monoclonal antibody that targets and neutralizes VEGF. It is currently being studied as an effective treatment in platinum-sensitive recurrent OC, although its efficacy in the treatment of BRCAm OC is unclear [84].

### Combined treatment

Combined therapeutic regimens are common in the treatment of BRCAm OC and the rationale behind this is that the use of different agents enables the targeting of distinct, non-redundant molecular mechanisms that lead to malignancy and tumor growth. Several studies have already characterized the use of different combinatorial treatment regimens, and many such studies are ongoing. Combined treatment is also considered the current standard of care because it can enable the overall lowering of systemic toxicity of the treatment regimen. An open-label, phase II, randomized study on 173 patients from 12 countries that aimed to assess the efficacy of chemotherapy alone (paclitaxel + carboplatin) or chemotherapy along with olaparib maintenance in recurrent OC found that the latter group showed an enhanced progression-free outlook, with positive outlook further increased in patients with BRCAm OC [61]. Olaparib and cediranib were investigated in a combination treatment regimen for HGSOC, and improved PFS rates were observed. However, any specific advantage in *BRCA*-linked HGSOC was not studied or reported as part of this investigation [63]. Recently, it has been shown in a phase I trial of olaparib in combination with bevacizumab that PARP inhibitors and anti-angiogenic agents have a complementary method of action and that the efficacy of PARP inhibition could increase because of the hypoxic environment induced by the anti-angiogenic treatment [85].

### Risk assessment and prevention of OC

Several studies have shown that cancers linked to *BRCA* mutations respond better to platinum-based chemotherapy and to PARP inhibitors and it is well established that *BRCA* mutation status can inform treatment. An observational study found that there were better survival rates in patients who tested for *BRCA* mutations and thus availed of therapies known to have better outcomes based on their genetic profiles [86]. Apart from improving treatment outcomes in patients who test for mutations after developing disease, hereditary screening for genetic *BRCA* mutations is a useful tool for the prevention of disease in patients with a known risk of developing certain malignancies. Various measures can be used to prevent the occurrence of OC in *BRCA* mutation carriers, including preventive surgery and the use of oral contraceptives [87, 88]. Preventive surgery, i.e., salpingo-oophorectomy (removal of the ovary and fallopian tubes), is used as a strategy for reducing the risk of OC occurrence in patients with known pathological *BRCA* mutations that predispose to OC. Several studies have shown that the risk of developing OC in patients with *BRCA* mutations can be significantly reduced, with one study reporting a decrease in the risk of OC occurrence of about 85% for patients who chose preventive surgery over surveillance programs as a strategy for risk reduction [89–91]. Currently, a phase II study is trying to evaluate both types of surgery, either RRSO (risk reducing salpingo-oophorectomy)or ISDO (interval salpingectomy with delayed oophorectomy), based on changes in sexual function, mental health, and quality of life (NCI-2016-00778, NCT02760849). Similar to the general population, studies have also shown that an increased number of anovulation periods during the lifetime of a woman are likely to reduce the risk of developing OC in *BRCA* mutation carriers, and oral contraceptives are used as a preventive strategy due to this fact [92–94]. In contrast, it was shown that stimulation of the ovaries by artificial methods such as IVF are likely to increase the risk of OC [95]. For those mutation carriers who do not accept risk reducing surgery, the use of oral contraceptives constitutes a viable alternative strategy which is supported by the results of extensive meta-analyses [87, 88]. One analysis of 3 studies reported that the risk of ovarian cancer decreased by almost 50% upon use of combined oral contraceptives. Interestingly, the results for risk reduction of breast cancer occurrence in *BRCA* mutation carriers were less convincing [87].

### Homologous recombination deficiency in BRCAm OC and beyond

Several studies have established the role of *BRCA1* and *BRCA2* in the repair of genetic lesions via the homologous recombination repair (HRR) pathway and tissues that lack the function of these proteins show a stunted DNA damage repair mechanism that is solely reliant on other molecular cascades [13]. Deficiencies in the function of these proteins can lead to the deployment of other repair pathways in the cell-like non-homologous end joining (NHEJ), which are error prone and function at a lower fidelity. This can cause an accumulation of mutations that ultimately result in malignancy (two hit hypothesis). Such tissues that lack a functional HRR pathway are termed homologous repair deficient (HRD). Reliance on alternative mechanisms makes all tumors that lack the HRR pathway highly sensitive to therapies such as PARP inhibitors. PARPi specifically target the functioning of alternate pathways that these cells rely on in order to repair genetic lesions [77]. A deficiency in the function of molecules other than *BRCA* 1/2 can also confer the phenotype of HRD. Because HRD was first identified as a phenotypic characteristic of *BRCA*-linked tumors, it is one of the characteristics of BRCA*ness*. Thus, the principle of synthetic lethality can be used to target such tissues. RAD50, RAD51, PALB2, and the components of the Fanconi anemia pathway (i.e., FANCA and FANCI) are examples of some such molecules [65, 96]. The Cancer Genome Atlas (TCGA) has reported that HRD could account for approximately 50% of all HGSOC cases [20]. Thus, current research is focused on establishing an HRD signature that can be used to test patient samples in future. This will help determine whether PARPi are likely to be an effective course of treatment depending on the specifics of the patient profile. It was recently shown that the myChoice companion diagnostics platform by Myriad Diagnostics can be used as an indicator of the inability of the tumor to repair DNA damage. This platform is capable of detecting loss of heterozygosity, telomeric allelic imbalance, and large-scale state transitions in tumor cells [97]. It was shown to be effective as a testing platform to predict patient responses to TESARO’s Niraparib, another oral PARP inhibitor which was shown to have positive effects on patient PFS independent of gBRCA mutation status when used as maintenance therapy [98]. Other PARP inhibitors are also under clinical evaluation. This includes Rucaparib, which showed encouraging results in a recent Phase II trial which evaluated dose dependent responses in patients with gBRCA mutated breast and ovarian cancer [99].

### Ongoing investigations

Several studies that aim to assess the efficacy and safety of existing treatments in BRCAm OC are currently ongoing. New molecules are also being developed to overcome hurdles that exist currently in the treatment of BRCAm OC. Of note among these are the recent class of cell cycle checkpoint inhibitors that are being developed for the treatment of BRCAm OC [100]. Studies that aim to assess different combinatorial treatment regimens are also being conducted. A summary of all these is presented in Table 2.

**Table 2:** Summary of ongoing clinical trials being conducted for the treatment of BRCAm OC

Trial Number	Phase	Indication	Class of Drug	Treatment	Sponsor
NCT01874353	III	PSR HGSOC	PARPi	Olaparib versus placebo	Astra Zeneca
NCT01844986	III	Advanced stage (FIGO III-IV) ovarian cancer	PARPi	Olaparib maintenance monotherapy versus placebo	Astra Zeneca
NCT01472783	II	PSR EOC	PARPi	Veliparib	Vejle Hospital and Abbott
NCT01445418	I	Recurrent OC	Chemotherapy + PARPi	Carboplatin + olaparib	National Cancer Institute
NCT00628251	II	Platinum-resistant BRCAm OC advanced BRCAm OC	PARPi	Dose titration of olaparib versus doxil	Astra Zeneca
NCT02282020	III	PSR OC	PARPi	Single agent chemotherapy versus olaparib	Astra Zeneca
NCT02326844	II	gBRCAm OC + disease progression post treatment with alternative PARPi	PARPi (second generation)	Talazoparib	National Cancer Institute
NCT00679783	II	BRCAm OC or recurrent high-grade OC	PARPi	Olaparib	Astra Zeneca
NCT01772979	II	Recurrent BRCAm OC and BRCA*ness* positive	DNA-binding agent (transcription blocking)	Trabectedin	Catholic University of the Sacred Heart
NCT00494442	II	Advanced stage *BRCA* 1/2 positive OC Failed prior chemotherapy	PARPi	Olaparib	Astra Zeneca
NCT02203513	II	BRCAm OC/HGSOC	Chk 1/2 inhibitor (second generation, inhibits cell cycle progression)	LY2606368	National Cancer Institute
NCT01661868	II	*BRCA* 1/2 positive recurrent OC Treated with alternative PARPi/no PARPi exposure	PARPi	Olaparib	Astra Zeneca
NCT01306032	II	Refractory *BRCA* 1/2-positive OC/HGSOC	Chemotherapy + PARPi	Metronomic oral cyclophosphamide + veliparib	National Cancer Institute
NCT01482715	II	g*BRCA*-positive OC	PARPi	Oral rucaparib	Clovis Oncology
NCT02855944	III	BRCAm OC	PARPi versus chemotherapy	Rucaparib vs chemotherapy	Clovis Oncology
NCT02476968	IV	PSR BRCAm OC	PARPi	Olaparib maintenance monotherapy	Astra Zeneca
NCT00892736	I	Refractory BRCAm OC/platinum-resistant OC	PARPi	Veliparib	National Cancer Institute
NCT01853306	I	*BRCA* 1/2 positive HGSOC	PARPi	Veliparib	AbbVie
NCT02286687	II	Somatic *BRCA* linked OC	PARPi (second generation)	Talazoparib	M.D. Anderson Cancer Center
NCT02345265	II	Recurrent BRCAm OC or HGSOC	PARPi + VEGFRi	Olaparib + cediranib maleate	National Cancer Institute
NCT02354586	II	BRCAm OC or HGSOC (received previous chemotherapy)	PARPi	Niraparib	Tesaro Inc.
NCT01237067	I	Refractory/Recurrent BRCAm OC	Chemotherapy + PARPi	Carboplatin + olaparib	National Cancer Institute
NCT01989546	I / II	Advanced stage BRCAm OC	PARPi	BMN 673	National Cancer Institute
NCT01540565	II	g*BRCA*-positive recurrent OC	PARPi	Veliparib	National Cancer Institute
NCT02482311	Ib	BRCAm OC refractory to PARPi treatment	Wee1 kinase inhibitor (inhibits cell cycle progression)	AZD1775	Astra Zeneca
NCT02470585	III	BRCAm OC of epithelial origin	Chemotherapy + PARPi	Carboplatin + paclitaxel + veliparib maintenance versus carboplatin + paclitaxel + placebo maintenance	AbbVie
NCT01286987	I	Advanced stage/recurrent BRCAm OC	PARPi	Talazoparib	Medivation Inc.
NCT02489006	II	BRCAm OC	PARPi	Neoadjuvant olaparib treatment (before surgery and chemotherapy)	University Health Network, Toronto

OC: Ovarian Cancer; BRCAm: mutations in Breast cancer, early onset genes; FIGO: Federation of Gynecology and Obstetrics; PSR HGSOC: Platinum Sensitive Recurrent High Grade Serous OC; PARPi: poly ADP ribose polymerase inhibitor; VEGFRi: Vascular Endothelial Growth Factor Receptor inhibitor.

### Conclusions and areas of future advancement

Ovarian cancer, primarily of epithelial origin, is one of the major tumor types that is associated with *BRCA* mutations. Among gynecological cancers, OC is the cause of significant morbidity in the female population. Testing for *BRCA* mutations is an important step in the risk assessment, treatment and prognosis of patients with OC, since BRCAm OC has been associated with distinct molecular and histopathological characteristics that result in differential responses to certain therapeutic regimens. However, the prevalence and type of *BRCA* mutations vary between countries/populations, ethnicity, and type of cancer. Despite the presence of several Consortiums and guidelines, screening for these mutations and annotating a specific clinical significance to them remain a challenge.

Although the presence of *BRCA* mutations represents a significant increase in the risk of occurrence of OC, BRCAm OC is known to be more responsive to certain types of treatments such as platinum-based chemotherapy and to targeted treatments which disrupt the DNA-damage response pathways of the cell, most notably PARP inhibitors such as Olaparib, Niraparib and Rucaparib. The recent approval of such targeted therapies for the treatment of BRCAm OCs is encouraging. However, an urgent need exists to solve the problem of increasing resistance to these compounds. Preliminary investigations have shown that combining PARPi with PI3K inhibitors constitutes an effective strategy which prevents resistance to treatments used for triple negative breast cancer. It has also been shown in mouse mammary tumors that the loss of 53BP1 is likely to cause resistance to PARP inhibitors [101, 102].

In addition, differences in response rates to treatments exist, depending on whether the OC is of *BRCA1* or *BRCA2* origin [41]. Such differences may also exist depending on which regions of these genes contain the mutation, and a limited number of studies have focused on characterizing these variations in response. A more detailed analysis of such differences can further improve the treatment course, quality of life, and overall survival rates in patients with BRCAm OC.

There is an increasing body of evidence to show that certain therapies such as PARPi, which were initially developed for the treatment of BRCAm OC, can be extended to treat a spectrum of malignancies that are not linked to *BRCA* mutations, but exhibit certain molecular characteristics in common with *BRCA*-associated disease, specifically HRD [97]. Such therapy has the potential to improve disease outcomes, not only in the restricted population of *BRCA* mutation carriers, but also for use in all tumors with HRD as a defining characteristic.
